# Colchicine Does Not Reduce Abdominal Aortic Aneurysm Growth in a Mouse Model

**DOI:** 10.1155/2022/5299370

**Published:** 2022-09-30

**Authors:** James Phie, Shivshankar Thanigaimani, Pacific Huynh, Raghuveeran Anbalagan, Corey S. Moran, Robert Kinobe, Joseph V. Moxon, Matt A. Field, Smriti M. Krishna, Jonathan Golledge

**Affiliations:** ^1^The Vascular Biology Unit, Queensland Research Centre for Peripheral Vascular Disease, James Cook University, Townsville, Queensland, Australia; ^2^School of Dentistry, The University of Queensland, Herston Campus, Brisbane, Queensland, Australia; ^3^College of Public Health, Medical & Veterinary Sciences, James Cook University, Townsville, Queensland, Australia; ^4^Australian Institute of Tropical Health & Medicine and Centre for Tropical Bioinformatics and Molecular Biology, James Cook University, Townsville, Queensland, Australia; ^5^Immunogenomics Lab, Garvan Institute of Medical Research, Darlinghurst, NSW, Australia; ^6^Menzies School of Health Research, Darwin, NT, Australia; ^7^Baker Department of Cardiometabolic Health, University of Melbourne, Melbourne, Victoria, Australia; ^8^Department of Vascular and Endovascular Surgery, Townsville University Hospital, Townsville, Queensland, Australia

## Abstract

**Background and Aims:**

The nacht domain, leucine-rich repeat, and pyrin domain-containing protein 3 (NLRP3) inflammasome is upregulated in human abdominal aortic aneurysm (AAA), but its pathogenic role is unclear. The aims of this study were firstly to examine whether the inflammasome was upregulated in a mouse model of AAA and secondly to test whether the inflammasome inhibitor colchicine limited AAA growth.

**Methods:**

AAA was induced in eight-week-old male C57BL6/J mice with topical application of elastase to the infrarenal aorta and oral 3-aminopropionitrile (E-BAPN). For aim one, inflammasome activation, abdominal aortic diameter, and rupture were compared between mice with AAA and sham controls. For aim two, 3 weeks after AAA induction, mice were randomly allocated to receive colchicine (*n* = 28, 0.2 mg/kg/d) or vehicle control (*n* = 29). The primary outcome was the rate of maximum aortic diameter increase measured by ultrasound over 13 weeks.

**Results:**

There was upregulation of NLRP3 markers interleukin- (IL-) 1*β* (median, IQR; 15.67, 7.11-22.60 pg/mg protein versus 6.87, 4.54-11.60 pg/mg protein, *p* = .048) and caspase-1 (109, 83-155 relative luminosity units (RLU) versus 45, 38-65 RLU, *p* < .001) in AAA samples compared to controls. Aortic diameter increase over 80 days (mean difference, MD, 4.3 mm, 95% CI 3.3, 5.3, *p* < .001) was significantly greater in mice in which aneurysms were induced compared to sham controls. Colchicine did not significantly limit aortic diameter increase over 80 days (MD -0.1 mm, 95% CI -1.1, 0.86, *p* = .922).

**Conclusions:**

The inflammasome was activated in this mouse model of AAA; however, daily oral administration of colchicine did not limit AAA growth.

## 1. Introduction

Surgical repair is the only current treatment for abdominal aortic aneurysms (AAA) [1]. In order to discover AAA drugs, a number of rodent models have been developed [1]. Previous animal studies have mainly used the angiotensin II, intraluminal elastase, or calcium chloride models [1]. These models simulate some but not all pathological characteristics of human AAA [1]. A major limitation of these models is that increase in aortic diameter is limited to 1-2 weeks meaning that they are not suited to testing the effect of drugs on growth of established aneurysms [1].

A novel mouse model in which AAA is induced by topical application of elastase to the infrarenal aorta (IRA) and oral administration of 3-aminopropionitrile fumarate salt (E-BAPN) has recently been reported [2]. AAAs in this model have been reported to grow slowly for at least 100 days [2]. Aneurysms have many features of human AAA including focal expansion of all layers of the aortic wall, intraluminal thrombus (ILT), vascular smooth muscle cell apoptosis, extracellular matrix degradation, and aortic wall infiltration by neutrophils, macrophages, and CD3^+^ T cells [2]. This model was therefore chosen as it has relevance to human AAA pathology and is uniquely suited to testing the long-term effects of drugs on aneurysm growth.

The nacht domain, leucine-rich repeat, and pyrin domain-containing protein 3 (NLRP3) inflammasome is a proinflammatory pathway that leads to activation of caspase-1, interleukin-1-beta (IL-1*β*), and interleukin-18 (IL-18) [3, 4]. NLRP3 expression has been reported to be upregulated in human AAA samples compared with aortic samples from patients with atherothrombosis [5]. Experimental studies have suggested the NLRP3 inflammasome as a potential therapeutic target to prevent AAA progression [6] [7]. The effect of NLRP3 inflammasome blockade on the progression of established AAA has not however been tested. Colchicine is an oral gout medication that accumulates in neutrophils and inhibits their infiltration into tissues [8, 9]. Colchicine prevents microtubule assembly, thereby inhibiting the inflammasome, limiting generation of leukotrienes, and blocking phagocytosis [10]. Colchicine has been shown to prevent cardiovascular events such as myocardial infarction in high risk patients and therefore considered as a potentially useful oral cardiovascular drug, but its role in treating AAA is unknown [11].

The aims of this study were firstly to test whether the NLRP3 inflammasome was upregulated in the E-BAPN AAA model and secondly to test whether colchicine reduced AAA growth in this model. We also assessed the genomic changes in small AAAs in order to identify treatment targets to slow AAA growth. We performed transcriptomic analyses of samples from day 7 after aneurysm induction which is reflective of the stage of established small AAA in the E-BAPN model.

## 2. Materials and Methods

### 2.1. Mice

Seven-week-old male C57BL/6J mice were purchased from the animal resources centre (Western Australia) and acclimatised for one week at the James Cook University animal facility. Mice were maintained on a 12 hour light/dark cycle, at a relative humidity of 55 ± 2% and a temperature of 23 ± 2°C. Prior to experiments, mice were maintained on normal laboratory chow and water *ad libitum.* Ethics approval was obtained from the James Cook University Animal Ethics Committee, and experiments were conducted according to the NHMRC Australian code for the care and use of animals for scientific purposes guidelines for animal care and maintenance (2013). Research was reported in accordance with the Animal Research: Reporting of In Vivo Experiments (ARRIVE) guidelines 2.0 [12]. Cotton, shredded paper, and cardboard tubes were provided as environmental enrichment for all mice.

### 2.2. Study Design

Sham and AAA induction surgeries were conducted when mice were eight weeks old [2]. Body weight was measured weekly. The study was divided into two aims.

For aim one, the E-BAPN AAA model was validated through two experiments. A short-term study was conducted where mice were culled 5 days after AAA induction with E-BAPN (*n* = 3) or sham surgery (*n* = 3), and the IRA was assessed for differences in gene expression with an RNA-sequencing analysis. In a longer-term study, mice were culled 90 days after AAA induction with E-BAPN (*n* = 29) or sham surgery (*n* = 13) to examine AAA growth, NLRP3 activity, and ILT formation (Supplementary figure 1 and 2).

For aim two, the effect of colchicine on gene expression and AAA growth was examined in two experiments. A short-term study was conducted, whereby 9 mice were randomly allocated to colchicine (*n* = 3, 0.2 mg/kg/d, Sigma, Victoria) or vehicle control (*n* = 3) for 7 days in order to examine acute effects of colchicine. Two days after commencing the administration of colchicine or vehicle, AAAs were induced and mice were culled 5 days later for RNA-sequencing analysis of aortic tissue (Supplementary figure 1 and 2). In a longer-term study, AAAs were induced in 60 mice, and these mice were randomly allocated 21 days later to receive colchicine (*n* = 28, 0.2 mg/kg/d) or vehicle control (*n* = 29) for a further 69 days (Supplementary figure 1 and 2).

### 2.3. Induction of AAA

The E-BAPN model was generated according to a published protocol [2]. Porcine pancreatic elastase type I (20 *μ*L, 10.3 mg/mL, Sigma, Victoria) was applied to the IRA, and BAPN (0.2% *w*/*v*, Sigma, Victoria) was administered in drinking water from two days before surgery until the experimental endpoint. AAA was defined by a maximum IRA diameter of >1.5 mm.

### 2.4. Intervention and Control Groups

For aim one, mice were randomly assigned to receive either sham or AAA induction surgery. For aim two, mice were randomly allocated to receive colchicine dissolved in distilled water at 0.2 mg/kg or vehicle (distilled water without colchicine) via daily gavage. The dose of colchicine was calculated using the formula [animal equivalent dose (mg/kg) = (human dose = 2.5 mg)/(K_m_ ratio = 12.3)] translated to 0.2 mg/kg for mice [13]. The body surface area (BSA) conversion index for a 20 g mouse was 0.0007 [13]. The human dose of 2.5 mg translated in mice to 0.002 mg of colchicine per day using the BSA conversion index. Since the weight of the mice in this study ranged between 25 and 30 g, we used the km ratio calculations translated to a higher dose of 0.006 mg of colchicine per day for mice weighing 30 g, which is a currently acceptable dose for use in the treatment of gout [14] and has been the most commonly used dose in randomized controlled trials assessing the effects of colchicine on cardiovascular disease [15]. The proposed dose is also similar to what has previously been used in mouse studies [16]. Randomisation was performed using a sequence created by a random number generator. Outcome assessors were blinded to group allocation during ultrasounds and all data analyses.

### 2.5. Measurement of Tissue IL-1*β* Concentration and Caspase-1 Activity

The primary outcome for aim one was IRA IL-1*β* concentration, which was measured using a fluorometric ELISA (Catalog number - ab229384, Abcam, Victoria) performed according to manufacturer's instructions. Relative caspase-1 activity was detected using a Caspase-Glo 1 Inflammasome Assay (Catalog number - G9951, Promega, NSW) according to the manufacturer's instructions and measured using a Polarstar Omega plate reader.

### 2.6. Ultrasound Assessment of IRA Diameter

The primary outcome for aim two was AAA growth measured over time from outer to outer wall in the anterior-posterior orthogonal plane during late systole using a 10 MHz linear ultrasound transducer probe (Esaote, Italy) attached to a MyLab 30 ultrasound machine (Esaote, Italy), as previously described [17]. Diameters were measured at baseline (day 0) and 7, 21, 28, 42, 63, and 80 days after AAA induction. The intraobserver and interobserver reproducibility of IRA diameter measurement was assessed from ten repeats, and the coefficients of variation (CoV) were 4.4% and 7.2%, respectively.

### 2.7. Other Methods

The methods used for assessment of AAA diameter by morphometry, rupture, aneurysm severity, histology, immunofluorescence, RT-PCR, and RNA-sequencing are included in supplementary materials.

### 2.8. Sample Size Calculations

The required sample size for aim one was estimated based on aortic tissue IL-1*β* reported in a previous study in the angiotensin II model (vehicle 32 ± 14 pg/100 *μ*g protein, AAA 164 ± 115 pg/100 *μ*g protein, mean ± standard deviation) [6]. Based on these values, at least 8 mice were required at the experimental endpoint to test if IL-1*β* was upregulated (80% power, alpha 0.05, two-tailed). For aim two, the sample size was based on testing the hypothesis that AAA growth would be 20% lower in mice receiving colchicine compared to vehicle control [18]. A previous study reported that control mice undergoing the same AAA induction surgery exhibited a mean increase in IRA diameter of 800 ± 160% after 100 days [2]. Assuming a similar outcome for the control group in the current study and equal variance in both groups, a minimum sample size of 17 mice per group would be required to test if colchicine reduced AAA growth (power 80%, alpha 0.05). Sample sizes were increased by 80% to account for potential losses during the longer-term experiments.

### 2.9. Statistical Analyses

Data were tested for normal distribution with D'agostino and Pearson's test. Data that were normally distributed were analysed using unpaired two-tailed *t*-tests and presented as means ± standard error of means (SEM), and data not normally distributed were analysed using Mann-Whitney *U* tests and presented as median with interquartile range (IQR) and individual values. Intraluminal thrombus (ILT) presence was compared using Fisher's exact test, and AAA rupture was compared using the Mantel-Cox (log-rank) test. AAA growth analyses included all ultrasound data obtained up until the point of death and was performed using random slope and random intercept linear mixed effects models. Group allocation was treated as a fixed effect; mouse and time were included as random effects. The test statistic was interaction of time and intervention. Model fit was assessed by examination of residual distribution and qq-norm plots. Where necessary, the response variables were log transformed to adhere to model assumptions of linearity.

### 2.10. Bioinformatics

Log transformed data were used to calculate *p* values in cases where it improved model fit; however, nonlog transformed values were reported to make data easier to interpret. RNA-sequencing FastQ files were aligned to reference genome GRCh38.p13 using STAR [19] and underwent quality control, normalisation, and differential expression analysis using the bioconductor consensus DE package [20]. Preranked Gene Set Enrichment Analyses (GSEA) were performed with log fold change values using gene ontology (GO) biological pathways (Baderlabs) up to date as of 1 September 2021, and the results were visualised as enrichment maps using *cytoscape 3.8.2* with enrichment map app. The genes were collapsed to human orthologs for all analyses and presented as gene symbols. Differences were considered statistically significant when *p* values were < .05, or when *q* values were < .1 for GSEA of RNA-sequencing data. Statistical analyses were performed using GraphPad Prism V.6, GSEA desktop 4.1.0, and R Studio 4.1.2.

## 3. Results

### 3.1. Characteristics of the E-BAPN Model

**Figure 1:**
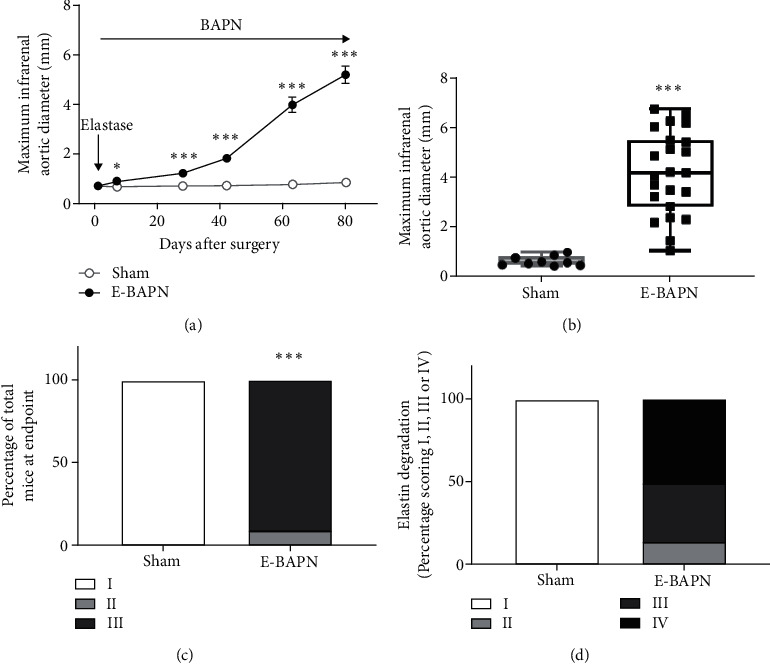
The effect of E-BAPN on infrarenal aortic diameter and structure. (a) IRA diameter in mice receiving E-BAPN and sham controls measured by ultrasound and AAA growth was assessed using linear mixed model analysis. Data were log transformed during statistical analysis to conform to model assumptions; however, raw values are shown. (b) Maximum IRA diameter in mice receiving E-BAPN and sham controls measured ex vivo at the experimental endpoint. Data were assessed as normally distributed and were analysed using unpaired two-tailed *t*-tests and presented in box plots. The box indicates the range between 1^st^ and 3^rd^ interquartile range. The whiskers indicate the minimum and maximum values. (c) Percentage of mice with different severity of aneurysm. I, no aneurysm; II, aortas between 150 and 300% of nondiseased controls; III, >300% of nondiseased controls. Data was analysed using Fischer's exact test. (d) Percentage of mice with different severity of aortic elastin degradation. I, no elastin degradation; II, mild fragmentation or damage; III, moderate damage; IV, severe fragmentation with sections of complete destruction of all elastic lamellae. Data were analysed using Fischer's exact test. Mice receiving E-BAPN had significantly higher AAA growth over 80 days (^∗∗∗^*p* < .001; ^∗^*p* < .050) and significantly higher aneurysm presence (*p* < .001). E-BAPN *n* = 23 and sham *n* = 10 on day 90. E-BAPN: elastase and 3-aminopropionitrile; IRA: infrarenal aorta.

Increase in IRA diameter determined by ultrasound was significantly greater in mice receiving E-BAPN compared with sham operated mice over an 80 day period (mean difference, MD, 4.3 mm, 95% CI 3.3, 5.3, *p* < .001). *Ex vivo* morphometry data at day 90 showed significantly greater IRA diameter in E-BAPN compared to sham operated mice (median, 4.18 mm, IQR, 2.84-5.49 versus 0.60 mm, 0.51-0.84, *p* < .001, Figures 1(a) and 1(b)). After 90 days, AAAs were present in all (100%) mice receiving E-BAPN, and no AAAs were observed in the sham mice (Figure 1(c) and Supplementary figure 3, *p* < .001). Aortic elastin degradation was present in all (100%) mice receiving E-BAPN including severe fragmentation and areas of complete destruction in 12 mice (50%); however, no elastin degradation was detected in sham mice (Figure 1(d)). Seventeen mice (23%) from the experimental groups died between the start of the experiment and sacrifice. Six mice receiving E-BAPN died due to aortic rupture, including three ruptures in the suprarenal aorta and three in the IRA. Eleven mice died due to causes unrelated to aneurysm rupture (Supplementary table 1). A summary of key features of the E-BAPN model compared to other AAA models are shown in supplementary table 2.

### 3.2. Genes Differentially Expressed between E-BAPN AAA Mouse Model versus Sham Mice after 7 Days

**Figure 2:**
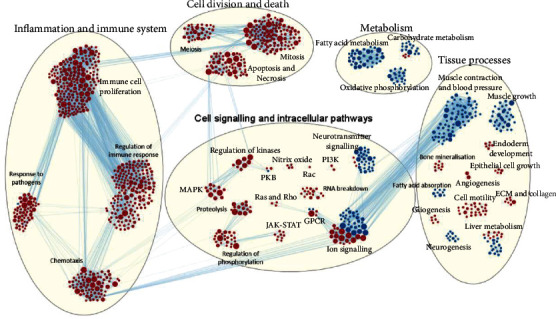
The effect of E-BAPN administration on gene expression and pathway regulation. (a) GSEA enrichment map showing pathways that are upregulated and downregulated in mice receiving E-BAPN compared with sham controls (*q* < .1). Red circles indicate upregulated pathways, and blue circles indicate downregulated pathways in mice receiving E-BAPN compared with sham controls. Circle size indicates the size of the gene set, and darker shades of red or blue indicate higher normalised enrichment score indicating more substantial pathway activation. Blue lines indicate overlap of differentially expressed genes involved in specific pathways. E-BAPN: elastase and 3-amniopropionitrile; GSEA: Gene Set Enrichment Analysis; ECM: extracellular matrix; GPCR: G-protein coupled receptor; JAK-STAT: Janus kinase-signal transducer and activator of transcription; MAPK: mitogen-activated protein kinase; PI3K: phosphoinositide 3-kinase; PKB: protein kinase B.

Data of 15,354 known genes, including 8221 that were differentially expressed between E-BAPN AAA mice and sham mice, were included in pathway analyses. Out of 14,214 listed biological pathways, 893 were significantly upregulated and 277 were significantly downregulated in E-BAPN AAA mice compared with sham controls (*q* < 0.1). The GSEA showed that the most substantially upregulated pathways were inflammatory and most downregulated pathways were related to muscle contraction (Figure 2 and supplementary table 3). Heat maps of overall gene expression and within the most upregulated pathway (positive regulation of leukocyte activation) showed upregulation of proinflammatory genes including those related to the NLRP3 (supplementary figure 4). The potent neutrophil chemoattractant and activator granulocyte chemotactic protein 2 (CXCL6; fold difference, FD: 10.35; *p* < 0.001) and IL-6 (FD: 8.32; *p* < 0.001) were the most highly differentially expressed proinflammatory genes (Supplementary figure 4). There was significant upregulation of inflammasome genes in AAA samples suggesting this pathway was an important target to limit growth of small AAAs (Supplementary table 4). In addition, there was a significant upregulation of 9 genes and significant downregulation of 6 genes expressed in myeloid cells in AAA samples compared to aortic samples from sham controls (Supplementary table 5).

### 3.3. IRA Caspase-1 Activity and IL-1*β* Concentration in E-BAPN AAA Mice Model versus Sham Mice after 90 Days

**Figure 3:**
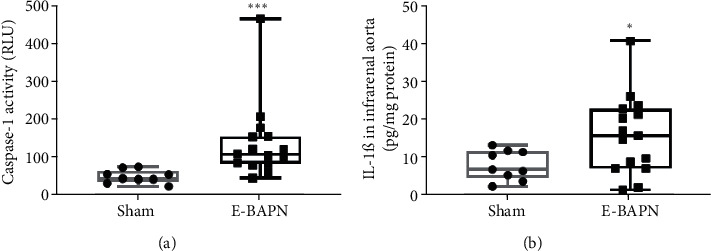
The effect of E-BAPN on the NLRP3 inflammasome. (a) Relative caspase-1 activity within the IRA of E-BAPN-treated mice and sham controls. (b) IL-1*β* protein concentration within the IRA of E-BAPN-treated mice and sham controls. E-BAPN-treated mice had significantly higher IRA caspase-1 activity (^∗∗∗^*p* < .001), and IL-1*β* concentration (^∗^*p* < .050) compared with sham controls. E-BAPN *n* = 15 and sham *n* = 9. RLU: relative luminosity units; E-BAPN: elastase and 3-amniopropionitrile; IL-1*β*: interleukin-1*β*; IRA: infrarenal aorta. Data were assessed as normally distributed and were analysed using unpaired two-tailed *t*-tests and presented in box plots. The box indicates the range between 1^st^ and 3^rd^ interquartile range. The whiskers indicate the minimum and maximum values.

Aortic caspase-1 activity (109, 83-155 relative luminosity units (RLU) versus 45, 38-64.5 RLU, *p* < .001, Figure 3(a)) and IL-1*β* concentration (15.67, 7.11-22.60 pg/mg protein versus 6.87, 4.54-11.60 pg/mg protein, *p* < .050, Figure 3(b)) were greater in aortic tissue samples of E-BAPN AAA mice compared with sham controls at day 90.

### 3.4. Effect of Colchicine in E-BAPN AAA Mice versus Vehicle Treatment on Growth of Established AAAs Assessed over 80 Days

**Figure 4:**
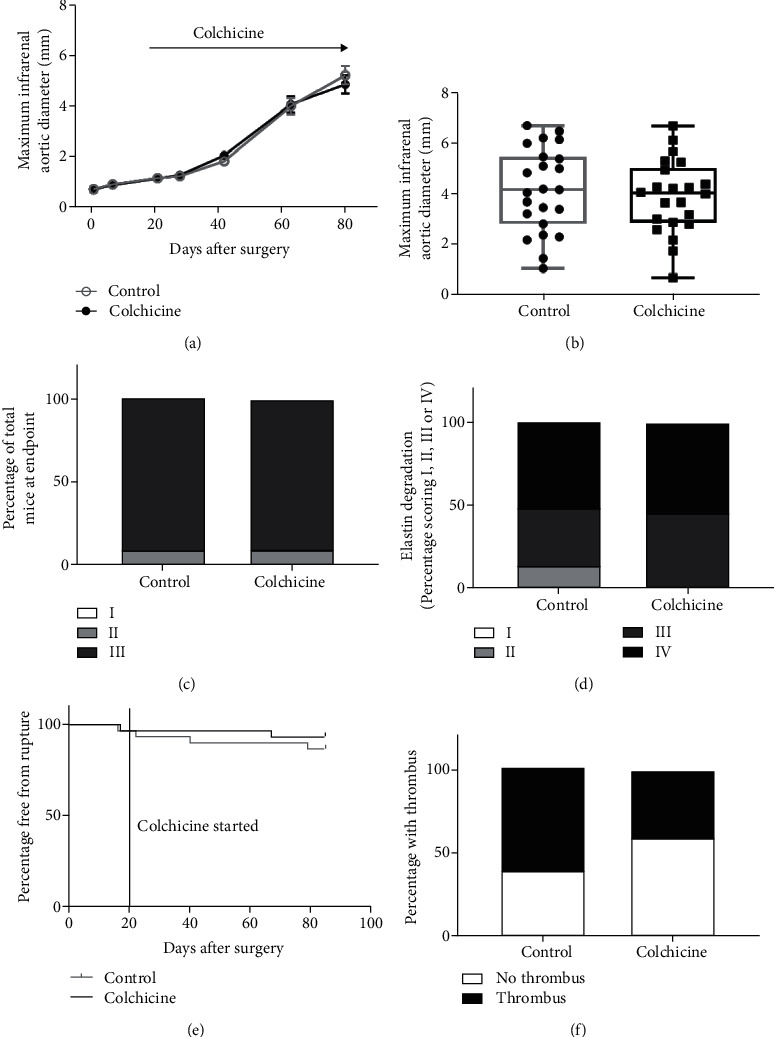
The effect of oral colchicine administration on AAA incidence, severity, and rupture rates. (a) Maximum IRA diameter measured by ultrasound and analysed using linear mixed effects modeling. Data were log transformed during statistical analysis to conform to model assumptions; however, raw values are shown. (b) Maximum diameter of the IRA as measured by morphometry at the end of the experiment (day 90). Data were assessed as normally distributed and were analysed using unpaired two-tailed *t*-tests and presented in box plots. The box indicates the range between 1^st^ and 3^rd^ interquartile range. The whiskers indicate the minimum and maximum values. (c) Percentage of mice with different severity of AAA. I, no aneurysm; II, 150-300% normal diameter; III, >300% normal diameter. Data were analysed using Fischer's exact test. (d) Percentage of mice with different severity of elastin degradation. I, no elastin degradation; II, mild fragmentation or damage; III, moderate damage; IV, severe fragmentation with sections of complete destruction of all elastic lamellae. Data was analysed using Fischer's exact test. (e) Percentage of mice free from aneurysm rupture using Kaplan-Meier's survival curve analysed using log-rank test. (f) Percentage of mice with and without ILT. Data were analysed using Fischer's exact test. Control *n* = 23 and colchicine *n* = 22 at day 90. IRA: infrarenal aorta.

Colchicine did not significantly reduce AAA growth over an 80-day period compared to vehicle control as measured by ultrasound (MD -0.1 mm, 95% CI -1.1, 0.86, *p* = .922, Figure 4(a)). There was no significant difference in ex vivo AAA diameters between mice receiving colchicine and mice receiving vehicle on day 90 (4.0 mm, 2.9-5.0 versus 4.2 mm, 2.8-5.5, *p* = .713, Figure 4(b)). AAAs were present in all mice and considered severe in 20 (90%) mice receiving colchicine compared with 21 (91%) mice in the control group on day 90 (Figure 4(c) and Supplementary figure 5). Elastin degradation was present in all aortic samples of mice receiving either colchicine or vehicle control, with severe fragmentation and areas of complete destruction in 12 (53%) mice receiving colchicine compared with 12 (50%) in the vehicle control mice at the endpoint (Figure 4(d)). Aneurysm rupture occurred in one mouse from the colchicine group (4%) and three mice from the control group (10%, *p* = .730, Figure 4(e)). Of mice reaching the experimental endpoint, ILT was present in 9 of 22 (41%) mice receiving colchicine and 14 of 23 (61%) control mice (Figure 4(f), *p* = .238).

### 3.5. Effect of Colchicine in E-BAPN AAA Mouse Model versus Vehicle Treatment on Inflammatory Pathway Expression in the IRA after 7 Days

Data of 15,354 known genes, including 620 that were differentially expressed between mice receiving colchicine and mice receiving vehicle control, were included in pathway analyses. Out of 14,214 listed biological pathways, one pathway, associated with oxidative demethylation, was significantly upregulated, and no pathways were downregulated (*q* < 0.1). The aortic expression of inflammasome genes was not downregulated in mice receiving colchicine by comparison to vehicle controls. These results suggest that colchicine did not block the inflammasome in the short term (Supplementary table 4).

### 3.6. Effect of Colchicine in E-BAPN AAA Mice versus Vehicle Treatment on Caspase-1 Activity and Inflammatory Cytokine Concentrations or RNA Expression after 90 Days

**Figure 5:**
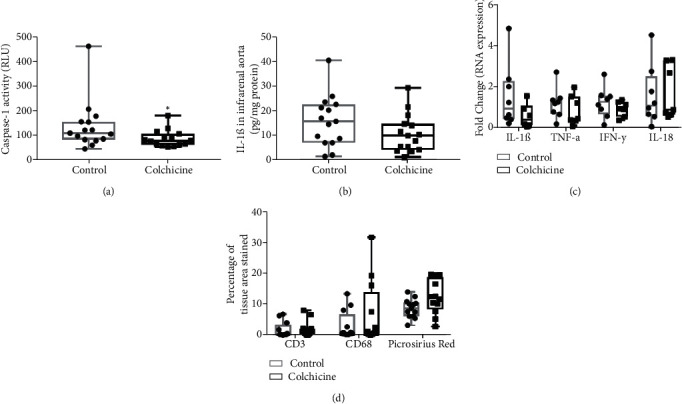
The effect of oral colchicine on tissue cytokines and inflammatory cell markers related to the NLRP3 inflammasome. (a) Relative caspase-1 activity within the IRA of mice receiving colchicine intervention compared with controls. (b) IL-1*β* protein concentration within the IRA of mice receiving colchicine intervention compared with controls. Control *n* = 15 and colchicine intervention *n* = 15. (c) IL-1*β*, TNF-*α*, IFN-*γ*, and IL-18 RNA expressions within the IRA in mice receiving colchicine compared with controls. Control *n* = 8 and colchicine intervention *n* = 7. Fold change indicates the number of folds the RNA expression was increased compared to sham operated mice. (d) Percentage of tissue stained with CD3, CD68, and picrosirius red birefringence (as markers of T cells, macrophages, and collagen, respectively). CD3: control *n* = 12 and colchicine intervention *n* = 13; CD68: control *n* = 12 and colchicine intervention *n* = 12; picrosirius red: control *n* = 13 and colchicine intervention *n* = 12. ∗Mice receiving colchicine had significantly lower IRA caspase-1 activity at experimental endpoint (*p* < .050). All data were assessed as normally distributed and were analysed using unpaired two-tailed *t*-tests and presented in box plots. The box indicates the range between 1^st^ and 3^rd^ interquartile range. The whiskers indicate the minimum and maximum values. IFN-*γ*: interferon-*γ*; IL-1*β*: interleukin-1*β*; IRA: infrarenal aorta; RLU: relative luminosity units; TNF-*α*: tumour necrosis factor-*α*.

IRA caspase-1 activity was significantly lower in mice receiving colchicine compared with vehicle control (79, 63-106 RLU versus 109, 83-155 RLU, *p* = .047, Figure 5(a)). IRA IL-1*β* protein concentration (10.03, 4.25-14.73 pg/mg protein versus 15.67, 7.11-22.60 pg/mg protein, *p* = .174) was not significantly reduced in mice receiving colchicine compared with vehicle control (Figure 5(b)). Similarly, IL-1*β* (0.63, 0.16-1.58 fold change versus 0.96, 0.45-2.30 fold change, *p* = .397), TNF-*α* (0.47, 0.37-1.55 versus 1.25, 0.71-1.45, *p* = .380), IFN-*γ* (0.96, 0.57-1.28 versus 1.27, 0.68-1.59, *p* = .346), and IL-18 (0.91, 0.67-3.29 versus 1.11, 0.60-2.52, *p* = .828) RNA expression were not significantly reduced in mice receiving colchicine compared with controls (Figure 5(c)). IRA percentage area stained for CD3 T cells (0.81, 0.24-2.05% versus 0.23, 0.10-3.37%, *p* = .406), CD68 macrophages (1.09, 0.44-13.94% versus 0.67, 0.40-6.68%, *p* = .478), and collagen (12.06, 8.31-18.69% versus 8.69, 6.08-10.59%, *p* = .068, Figure 5(d)) were not significantly different between mice receiving colchicine and mice receiving vehicle control.

## 4. Discussion

This study suggests that the E-BAPN model is an effective new model for studying the longer-term effects of drugs on AAA growth [2]. This model exhibits all key characteristics of human AAA that the three main other mouse models do not (Supplementary table 2). In this study, we showed, like other AAA mouse models and in patients, that activity of the NLRP3 inflammasome mediator caspase-1 was elevated with concomitant increases in IL-1*β* concentrations within aneurysmal tissue [21]. This activation of the inflammasome was demonstrated in both short-term transcriptomic analyses and long-term biochemical analyses using aortic samples. Transcriptomic analyses using aortic samples also showed significant upregulation of other inflammatory pathways within the aortas of the E-BAPN model in keeping with prior research implicating inflammation in AAA pathogenesis (Supplementary table 3) [22, 23].

Treatment with colchicine for 80 days significantly reduced caspase-1 activity, a marker of NLRP3 inflammasome activity; however, this did not result in decreased aortic IL-1*β* concentration and did not significantly reduce AAA growth or rupture. Colchicine did not effectively block the upregulation of aortic inflammation in the short term based on the transcriptomic analyses suggesting that the blockade of inflammasome takes place over long-term treatment.

ILT is a consistent feature of human AAA and ILT presence, and size has been correlated with early rupture and increased AAA growth rates [24, 25]. Over half of control mice (61%) had ILT present, and these ILT had high abundance of CD3 T cells and CD68 macrophages compared to surrounding aortic tissue, similar to that reported for human AAA [26]. The occurrence of ILT and relative CD3 T cell and CD68 macrophage accumulation was not significantly different between mice receiving colchicine and vehicle control in the current study.

Low-dose colchicine (0.5-1.0 mg/day, comparable to current study based on body surface area translation) has been shown to reduce cardiovascular events in patients with coronary heart disease [11]. The dose of colchicine used in this study was chosen to mimic the low dose used in clinical trials and to minimise side effects and did not significantly reduce tissue IL-1*β* concentrations. Colchicine had a small effect on caspase-1 activity and significantly upregulated the oxidative demethylation pathway which appears to be related to colchicine metabolism [27]. A higher dose of colchicine could have potentially suppressed IL-1*β* concentrations in the long term and inflammasome in the short term which could have had different effects on AAA growth. However, higher doses of colchicine may not be feasible to translate to patients as a long-term treatment to limit AAA growth. The documented poor tolerance of patients to higher colchicine doses limits the clinical relevance of testing this [28].

The study design and AAA model used in this study have a number of strengths. Aneurysms produced by this methodology are true aortic dilatations as opposed to dissecting false aneurysms and share key features of the human disease including involvement of the IRA, presence of ILT, T cells and macrophages, decreased vascular smooth muscle cells and collagen deposition within the aortic wall, and increased risk of rupture [2, 26, 29]. The design of the current study also simulates the clinical disease, whereby colchicine was administered 21 days after aneurysms were established. The use of BAPN inhibits crosslinking of elastin and collagen, and currently, no intervention has been shown to limit AAA growth in this model [2, 30]. This is reflective of the negative findings of clinical trials, and it is possible that this model is more realistic to identify translatable therapies although this remains to be confirmed [1]. Limitations of this study should also be acknowledged. Colchicine did not effectively block all elements of the inflammasome. We based the dose of colchicine used in this study on that found to downregulate proinflammatory cytokines, limit the NLRP3 inflammasome, improve cardiac function, and increase survival after myocardial infarction in a past study [16]. The dose used was also calculated to be equivalent to that which has been shown to be safe and well tolerated in patients, effective in limiting cardiovascular events and used as a treatment for gout [14]. Transcriptomic analyses showed that a number of inflammasome-associated genes were significantly differentially expressed in the aneurysm model compared to sham controls, and the major over- and underexpressed genes are reported in supplementary table 4. However, colchicine only downregulated caspase-1 in the long term but not short term when compared to vehicle controls.

In conclusion, the E-BAPN model shows evidence of NLRP3 inflammasome activation. Despite this, administration of colchicine, an inhibitor of the NLRP3 inflammasome, did not limit AAA growth or inflammation observed in the E-BAPN model.

## Data Availability

Data are deposited in the repository maintained by James Cook University. It will be available on request after approval from the university.

## Supplementary Materials

Supplementary Materials Supplementary figure 1: experimental plan of all 6 groups included in the two experiments, including timing of surgery and intervention. Supplementary figure 2: modified consort flow diagram of mice included in the short-term and longer-term studies. Supplementary figure 3: photographs of aortas at study endpoint on day 90. (A) Aortas from sham mice. (B) Aortas from E-BAPN mice. Supplementary figure 4: heat maps of the most highly differentially expressed genes in sham and E-BAPN IRA on day 5. (A) Heat map of the top 10 most over and under expressed genes overall. (B) Heat map of the top 10 most over- and underexpressed genes in the positive regulation of leukocyte activation pathway. NLRP3 is highly differentially expressed indicating activation of the NLRP3 inflammasome on day 5. Supplementary figure 5: photographs of aortas at study endpoint on day 90. (A) Aortas from mice receiving E-BAPN and vehicle control. (B) Aortas from mice receiving E-BAPN and colchicine intervention. Supplementary figure 6: representative immunofluorescence images of control, colchicine treatment, and sham groups. Supplementary table 1: deaths during experimental period. Supplementary table 2: representation of the key features of human AAA that are present in animal models. Supplementary table 3: top 10 most upregulated and downregulated GO biological pathways in mice receiving E-BAPN compared with sham controls determined by GSEA. Supplementary table 4: table showing the inflammasome-associated genes in vehicle and colchicine IRA on day 7 compared to vehicle-treated mice. Supplementary table 5: the aortic expression of myeloid cell associated genes in mice receiving vehicle control and colchicine for 7 days by comparison to vehicle controls.
