# Supplementary material for: Colchicine Does Not Reduce Abdominal Aortic Aneurysm Growth in a Mouse Model
Source: Cardiovasc Ther. 2022 Sep 30;2022:5299370. doi: 10.1155/2022/5299370 (PMC9553691; doi:10.1155/2022/5299370)
Supplement: Supplementary Materials — Supplementary figure 1: experimental plan of all 6 groups included in the two experiments, including timing of surgery and intervention. Supplementary figure 2: modified consort flow diagram of mice included in the short-term and longer-term studies. Supplementary figure 3: photographs of aortas at study endpoint on day 90. (A) Aortas from sham mice. (B) Aortas from E-BAPN mice. Supplementary figure 4: heat maps of the most highly differentially expressed genes in sham and E-BAPN IRA on day 5. (A) Heat map of the top 10 most over and under expressed genes overall. (B) Heat map of the top 10 most over- and underexpressed genes in the positive regulation of leukocyte activation pathway. NLRP3 is highly differentially expressed indicating activation of the NLRP3 inflammasome on day 5. Supplementary figure 5: photographs of aortas at study endpoint on day 90. (A) Aortas from mice receiving E-BAPN and vehicle control. (B) Aortas from mice receiving E-BAPN and colchicine intervention. Supplementary figure 6: representative immunofluorescence images of control, colchicine treatment, and sham groups. Supplementary table 1: deaths during experimental period. Supplementary table 2: representation of the key features of human AAA that are present in animal models. Supplementary table 3: top 10 most upregulated and downregulated GO biological pathways in mice receiving E-BAPN compared with sham controls determined by GSEA. Supplementary table 4: table showing the inflammasome-associated genes in vehicle and colchicine IRA on day 7 compared to vehicle-treated mice. Supplementary table 5: the aortic expression of myeloid cell associated genes in mice receiving vehicle control and colchicine for 7 days by comparison to vehicle controls. [file 5299370.f1.docx]

**Colchicine does not reduce abdominal aortic aneurysm growth in a mouse model**

Supplementary materials

**Detailed Methods**

## Morphometric assessment of infrarenal aortic diameter

*Ex vivo* morphometry measurements of maximum IRA diameter were taken from outer to outer layer using photos and ImageJ analysis software (National Institutes of Health). The intra-observer and inter-observer reproducibility for *ex vivo* IRA measurements were calculated. The CoV were 6.0% and 10.5% (n=10) respectively.

**Aneurysm rupture**

Aneurysm ruptures were validated by post mortem and reported based on time of occurrence in the study and location that the rupture occurred.

**Aneurysm severity**

Aneurysm severity was graded using a modified version of a previously described system as: I) No aneurysm: Maximum IRA diameters below 150% of average non-diseased controls; II) Small aneurysms: Maximum IRA diameters between 150-300% of non-diseased controls; III) Maximum IRA diameters >300% of non-diseased controls ^1^.

**Histological assessment of elastin and collagen**

Frozen sections of the IRA were stained with Verhoeff van Giesons and picrosirius red as previously described ^2^. Severity of elastin degradation was graded according to the following criteria: I) no elastin degradation; II) mild fragmentation; III) moderate fragmentation; IV) severe fragmentation with sections of complete destruction of all elastic lamellae ^3^. The total area of picrosirius staining (collagen content) was expressed as a percentage of total IRA area on the slide (area of collagen staining/total tissue area). This percentage was determined for four to five fields of at least three sections from each mouse, and the mean value was calculated. We have previously reported good intra and inter-observer reproducibility using these techniques ^2, 4^.

**RNA-sequencing and RT-PCR**

RNA extraction was performed using an RNeasy mini kit (Qiagen, Victoria) according to manufacturer’s instructions. For RNA-sequencing, extracted samples were prepared using Illumina stranded total RNA kit with Ribo-zero Plus kit for ligation, and run on a NovaSeq 6000 SP 2x100bp flowcell. The average read pairs per sample were 60 million, and all samples passed quality control with an average RIN of 8.7 in the samples tested.

One-step RT-PCR (Qiagen, Victoria) was performed to determine gene expression of key cytokines of interest (IL-1β, TNF-α, IFN- γ, and IL-18) as previously described ^5^.

**Immunofluorescence**

Frozen sections were incubated with anti-mouse CD3 or CD68 primary antibodies (Abcam, Victoria) and visualised with Alexa-fluor 488 conjugated secondary antibodies (Abcam, Vic) based on previously described protocols ^6^. The total area of Alexa-fluor 488 staining was expressed as a percentage of total IRA area on the slide (area of CD3 or CD68 staining/total tissue area). We have previously reported good intra and inter-observer reproducibility using this technique ^7^.


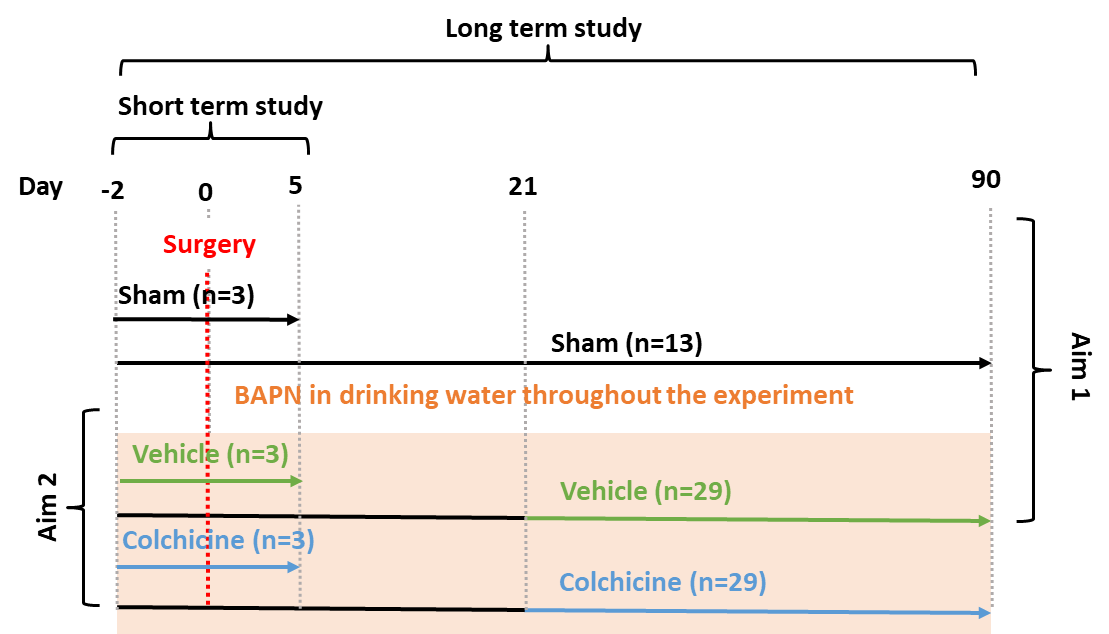


Supplementary figure 1. Experimental plan of all 6 groups included in the two experiments, including timing of surgery and intervention.


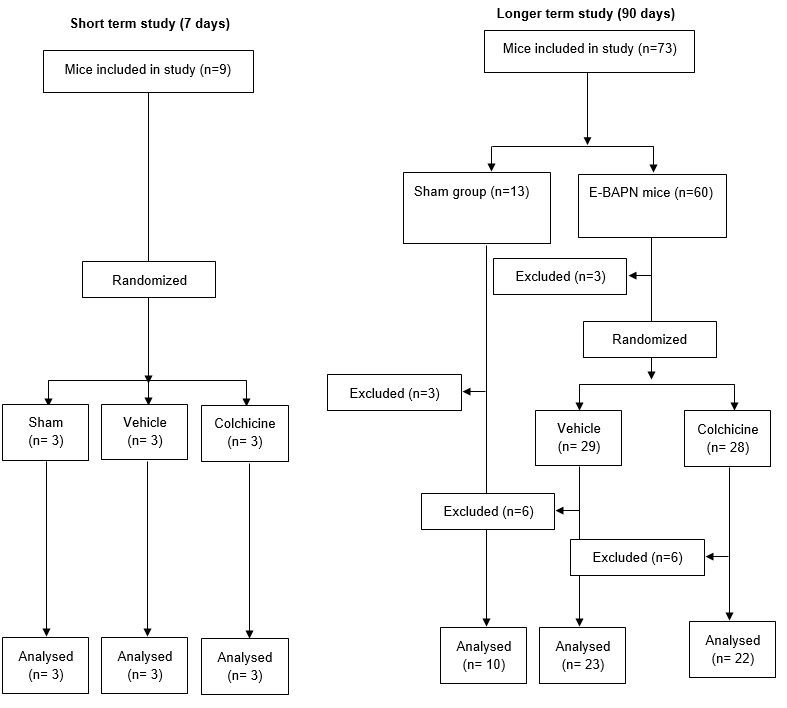


Supplementary figure 2. Modified consort flow diagram of mice included in the short term and longer term studies. Data from mice that had been obtained prior to exclusion was included in all analyses (modified intention to treat). All missing data were due to euthanasia due to ethical reasons, or death, with the exception of one mouse from the sham operated group where IRA tissue was not retrievable for endpoint analyses but complete ultrasound data were retrieved up until and including day 80.


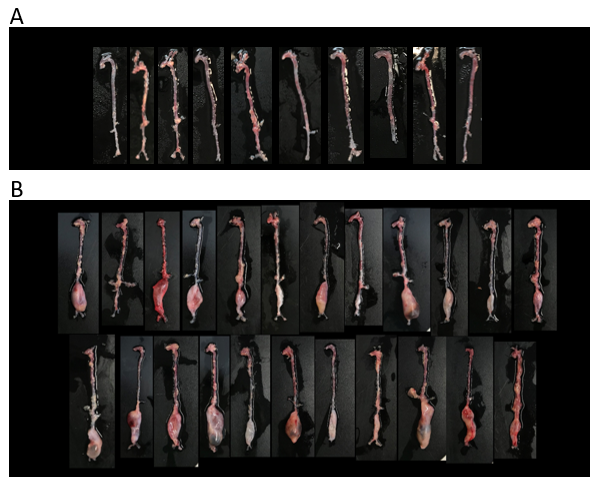


**A) Sham mice**

**B) E-BAPN mice**

Supplementary figure 3. Photographs of aortas at study endpoint on day 90. A) Aortas from sham mice. B) Aortas from E-BAPN mice. E-BAPN, elastase and 3-aminopropionitrile.


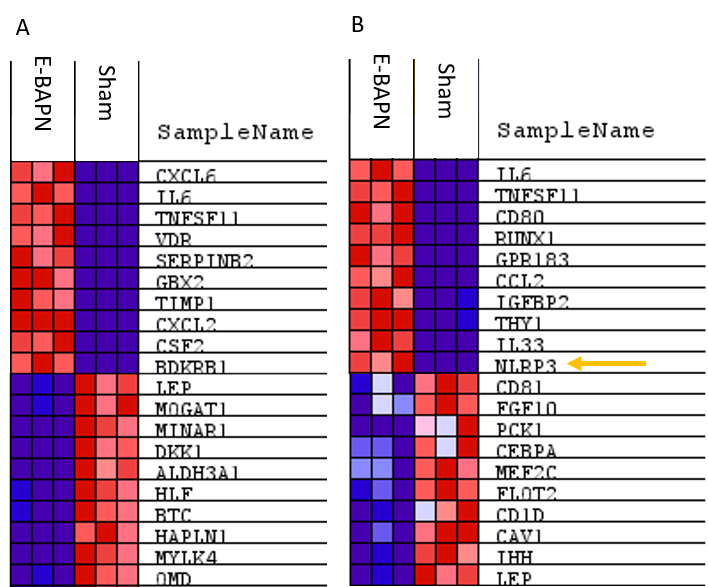


Supplementary figure 4. Heat maps of the most highly differentially expressed genes in sham and E-BAPN IRA on day 5. A) Heat map of the top 10 most over and under expressed genes overall. B) Heat map of the top 10 most over and under expressed genes in the positive regulation of leukocyte activation pathway. NLRP3 is highly differentially expressed indicating activation of the NLRP3 inflammasome on day 5 (orange arrow). E-BAPN, elastase and 3-aminopropionitrile; IRA, infrarenal aorta; NLRP3, nacht domain, leucine-rich repeat, and pyrin domain-containing protein 3.


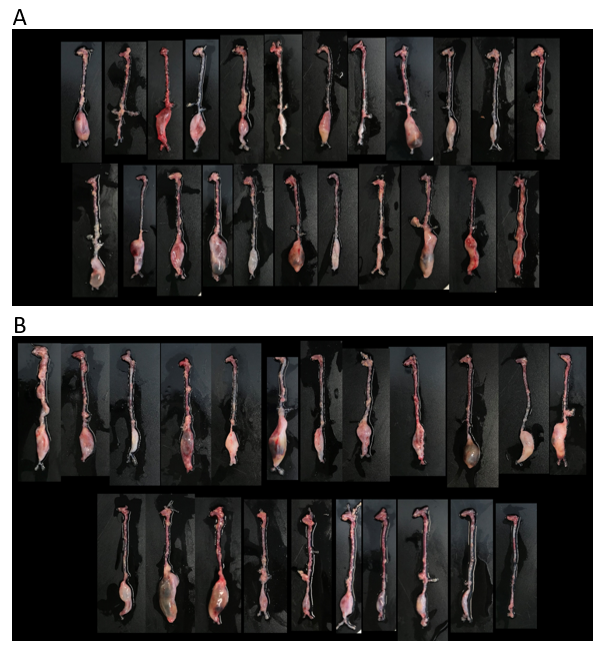


Supplementary figure 5. Photographs of aortas at study endpoint on day 90. A) Aortas from mice receiving E-BAPN and vehicle control. B) Aortas from mice receiving E-BAPN and colchicine intervention. E-BAPN, elastase and 3-aminopropionitrile.


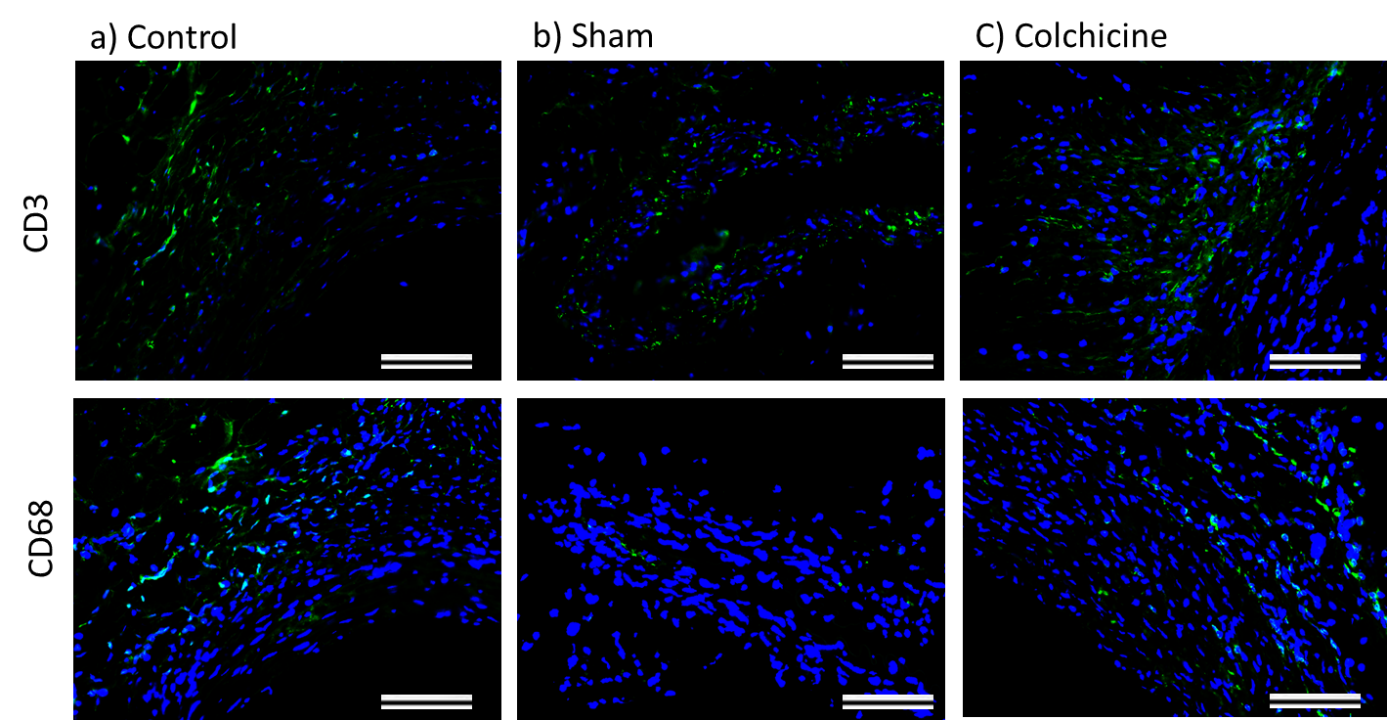


Supplementary figure 6: Representative immunofluorescence images of control, colchicine treatment and sham groups. Scale bar: 10μm.

Supplementary table 1. Deaths during experimental period

| **Day of death** | **Group** | **AAA diameter (mm)** | **Rupture site** |
| --- | --- | --- | --- |
| 6 | Not assigned | 0.7 |  |
| 16 | Not assigned | 1.1 | Infrarenal |
| 17 | Not assigned | 1.4 | Suprarenal |
| 22 | E-BAPN | 1.6 | Suprarenal |
| 23 | E-BAPN | 1.6 |  |
| 40 | E-BAPN | 4.4 | Infrarenal |
| 59 | Colchicine | 4.6 |  |
| 61 | Colchicine | 1.6 |  |
| 62 | Colchicine | 2.5 |  |
| 64 | Colchicine | 4.7 |  |
| 67 | Colchicine | 5.6 | Infrarenal |
| 74 | E-BAPN | 4.5 |  |
| 77 | E-BAPN | 4.0 |  |
| 79 | E-BAPN | 5.1 | Suprarenal |
| 80 | Sham | 0.7 |  |
| 81 | Sham | 0.7 |  |
| 82 | Colchicine | 5.7 |  |

Values measured from *ex vivo* images of dissected aortas. AAA, abdominal aortic aneurysm; E-BAPN, elastase and 3-aminopropionitrile.

Supplementary table 2. Representation of the key features of human AAA that are present in animal models.

| **Model** (Strain) | **Late Rupture** | **>28 days aneurysm growth** | **ILT** | **Elastin fragmentation** | **IRA aneurysm** | **True aneurysm** |
| --- | --- | --- | --- | --- | --- | --- |
| Ang II  (ApoE^-/-^) | N (early) | Y (<84 days) | N | Y | N | N |
| Elastase perfusion  (C57BL/6J) | N | N | Y (small) | Y | Y | Y |
| Calcium phosphate/ calcium chloride  (C57BL/6J) | N | N | N | Y | Y | Y |
| E-BAPN*  (C57BL/6J) | Y | Y (>90 days) | Y (large) | Y | Y | Y |

AAA, abdominal aortic aneurysm; Ang-II, Angiotensin-II; ApoE^-/-^, Apolipoprotein E deficient mouse; E-BAPN, topical aortic elastase and oral 3-aminopropionitrile; ILT, intraluminal thrombus; IRA, infrarenal aorta; Y, yes; N, no. Y answers are representative of human AAA ^8^.

Supplementary table 3. Top 10 most upregulated and downregulated GO biological pathways in mice receiving E-BAPN compared with sham controls determined by GSEA.

| # | GO biological pathway | Change | NES | q-value |
| --- | --- | --- | --- | --- |
| 1 | Positive regulation of leukocyte activation (GO:0002696) | Upregulated | 2.90 | <.001 |
| 2 | Regulation of lymphocyte activation (GO:0051249) | Upregulated | 2.90 | <.001 |
| 3 | Regulation of leukocyte activation (GO:0002694) | Upregulated | 2.88 | <.001 |
| 4 | Positive regulation of cell activation (GO:0050867) | Upregulated | 2.85 | <.001 |
| 5 | Regulation of t cell activation (GO:0050863) | Upregulated | 2.83 | <.001 |
| 6 | Positive regulation of lymphocyte activation (GO:0051251) | Upregulated | 2.82 | <.001 |
| 7 | Positive regulation of leukocyte cell-cell adhesion (GO:1903039) | Upregulated | 2.82 | <.001 |
| 8 | Regulation of cell activation (GO:0050865) | Upregulated | 2.81 | <.001 |
| 9 | Regulation of leukocyte cell-cell adhesion (GO:1903037) | Upregulated | 2.79 | <.001 |
| 10 | Positive regulation of t cell activation (GO:0050870) | Upregulated | 2.78 | <.001 |
| 1 | Muscle contraction (GO:0006936) | Downregulated | -2.57 | <.001 |
| 2 | Myofibril assembly (GO:0030239) | Downregulated | -2.53 | <.001 |
| 3 | Striated muscle cell development (GO:0055002) | Downregulated | -2.53 | <.001 |
| 4 | Muscle cell development (GO:0055001) | Downregulated | -2.51 | <.001 |
| 5 | Striated muscle contraction (GO:0006941) | Downregulated | -2.48 | <.001 |
| 6 | Cellular component assembly involved in morphogenesis (GO:0010927) | Downregulated | -2.48 | <.001 |
| 7 | Regulation of blood circulation (GO:1903522) | Downregulated | -2.47 | <.001 |
| 8 | Muscle system process (GO:0003012) | Downregulated | -2.46 | <.001 |
| 9 | Regulation of heart contraction (GO:0008016) | Downregulated | -2.42 | <.001 |
| 10 | Heart contraction (GO:0060047) | Downregulated | -2.41 | <.001 |

Data organised by NES. E-BAPN, elastase and 3-aminopropionitrile; GSEA, gene-set enrichment analysis; GO, gene ontology; NES, normalised enrichment score.

Supplementary table 4: Table showing the inflammasome associated genes in vehicle and Colchicine IRA on day 7 compared to vehicle treated mice.

| Gene ID | Gene name (symbol) | Sham vs. Vehicle | | Vehicle vs. Colchicine | |
| --- | --- | --- | --- | --- | --- |
|  |  | Mean FD±SD | p value | Mean FD±SD | p value |
| ENSMUSG00000037860 | absent in melanoma 2 (Aim2) | 2.96±0.04 | <0.001 | -0.06±0.07 | 0.922 |
| ENSMUSG00000032691 | NLR family, pyrin domain containing 3 (NLRP3) | 4.19±0.08 | 0.001 | -0.42±0.07 | 0.585 |
| ENSMUSG00000078942 | NLR family, apoptosis inhibitory protein 6 (NAIP6) | 2.33±0.04 | 0.001 | -0.14±0.07 | 0.774 |
| ENSMUSG00000039193 | NLR family, CARD domain containing 4 (NLRC4) | 2.21±0.04 | 0.001 | -0.15±0.07 | 0.778 |
| ENSMUSG00000078945 | NLR family, apoptosis inhibitory protein 2 (NAIP2) | 2.76±0.04 | 0.002 | -0.05±0.07 | 0.917 |
| ENSMUSG00000030921 | tripartite motif-containing 30A (TRIM30a) | 2.84±0.07 | 0.003 | -0.05±0.08 | 0.883 |
| ENSMUSG00000024079 | eukaryotic translation initiation factor 2-alpha kinase 2 (EIF2AK2) | 1.51±0.03 | 0.004 | -0.07±0.07 | 0.895 |
| ENSMUSG00000030793 | PYD and CARD domain containing (PYCARD) | 1.71±0.03 | 0.006 | -0.19±0.07 | 0.726 |
| ENSMUSG00000025888 | caspase 1 (CASP1) | 1.20±0.05 | 0.023 | 0.03±0.08 | 0.968 |

FD= Fold difference; SD=Standard deviation

Supplementary table 5: The aortic expression of myeloid cell associated genes in mice receiving vehicle control and colchicine for 7 days by comparison to vehicle controls.

| Gene ID | Gene name (symbol) | Sham vs. Vehicle | | Vehicle vs. Colchicine | |
| --- | --- | --- | --- | --- | --- |
|  |  | Mean FD±SD | p value | Mean FD±SD | p value |
| ENSMUSG00000042265 | Triggering receptor expressed on myeloid cells 1 (TREM1) | 7.05±0.29 | <0.001 | -0.66±0.07 | 0.533 |
| ENSMUSG00000041754 | Triggering receptor expressed on myeloid cells 3 (TREM3) | 5.50±0.20 | <0.001 | -0.86±0.07 | 0.835 |
| ENSMUSG00000090272 | Myeloid nuclear differentiation antigen like (MNDAL) | 2.26±0.06 | <0.001 | -0.07±0.07 | 0.877 |
| ENSMUSG00000053338 | T cell-interacting, activating receptor on myeloid cells 1 (TARM1) | 6.47±0.07 | <0.001 | -0.35±0.20 | 0.828 |
| ENSMUSG00000048416 | Myeloid leukemia factor 1 (MLF1) | -1.78±0.04 | <0.001 | 0.23±0.07 | 0.563 |
| ENSMUSG00000051682 | Triggering receptor expressed on myeloid cells-like 4 (TREML4) | 2.77±0.04 | <0.001 | -0.44±0.07 | 0.662 |
| ENSMUSG00000038612 | Myeloid cell leukemia sequence 1 (MCL1) | 0.62±0.04 | 0.001 | -0.58±0.07 | 0.006 |
| ENSMUSG00000071068 | Triggering receptor expressed on myeloid cells-like 2 (TREML2) | 2.72±0.18 | <0.001 | -0.09±0.10 | 0.906 |
| ENSMUSG00000019579 | Myeloid derived growth factor (MYDGF) | 0.61±0.04 | 0.004 | 0.07±0.08 | 0.623 |
| ENSMUSG00000053192 | Myeloid/lymphoid or mixed-lineage leukemia; translocated to, 11 (MLT11) | -0.84±0.07 | 0.003 | 0.06±0.08 | 0.804 |
| ENSMUSG00000023992 | Triggering receptor expressed on myeloid cells 2 (TREM2) | 1.50±0.23 | 0.021 | -0.59±0.08 | 0.432 |
| ENSMUSG00000025141 | Myeloid-associated differentiation marker-like 2 (MYADML2) | -2.95±0.29 | 0.002 | 0.18±0.09 | 0.892 |
| ENSMUSG00000030120 | Myeloid leukemia factor 2 (MLF2) | -0.25±0.04 | 0.002 | 0.05±0.08 | 0.283 |
| ENSMUSG00000028496 | Myeloid/lymphoid or mixed-lineage leukemia; translocated to, 3 (MLLT3) | -0.43±0.04 | 0.083 | 0.31±0.08 | 0.201 |
| ENSMUSG00000038437 | Myeloid/lymphoid or mixed-lineage leukemia; translocated to, 6 (MLLT6) | -0.23±0.04 | 0.118 | 0.11±0.08 | 0.416 |
| ENSMUSG00000023993 | Triggering receptor expressed on myeloid cells-like 1 (TREML1) | -2.78±0.86 | 0.005 | -2.11±0.21 | 0.354 |

FD= Fold difference; SD=Standard deviation

**References:**

[1] Lu, G, Su, G, Davis, JP, et al., A novel chronic advanced stage abdominal aortic aneurysm murine model, J. Vasc. Surg., 2017;66:232-242.e234.

[2] Krishna, SM, Moxon, JV, Jose, RJ, et al., Anionic nanoliposomes reduced atherosclerosis progression in Low Density Lipoprotein Receptor (LDLR) deficient mice fed a high fat diet, In, J. Cell. Physiol., J Cell Physiol, 2018.

[3] Krishna, SM, Seto, SW, Jose, RJ, et al., A peptide antagonist of thrombospondin-1 promotes abdominal aortic aneurysm progression in the angiotensin II-infused apolipoprotein-E-deficient mouse, Arterioscler Thromb Vasc Biol, 2015;35:389-398.

[4] Wang, Y, Krishna, SM, Moxon, J, et al., Influence of apolipoprotein E, age and aortic site on calcium phosphate induced abdominal aortic aneurysm in mice, Atherosclerosis, 2014;235:204-212.

[5] Biros, E, Walker, PJ, Nataatmadja, M, et al., Downregulation of transforming growth factor, beta receptor 2 and Notch signaling pathway in human abdominal aortic aneurysm, Atherosclerosis, 2012;221:383-386.

[6] Krishna, SM, Seto, SW, Jose, RJ, et al., A peptide antagonist of thrombospondin-1 promotes abdominal aortic aneurysm progression in the angiotensin II–infused apolipoprotein-E–deficient mouse, Arterioscler. Thromb. Vasc. Biol., 2015;35:389-398.

[7] Golledge, J, Cullen, B, Rush, C, et al., Peroxisome proliferator-activated receptor ligands reduce aortic dilatation in a mouse model of aortic aneurysm, Atherosclerosis, 2010;210:51-56.

[8] Golledge, J, Krishna, SM and Wang, Y, Mouse models for abdominal aortic aneurysm, Br. J. Pharmacol., 2020.
